# Long Non-coding RNAs Involved in Pathogenic Infection

**DOI:** 10.3389/fgene.2020.00454

**Published:** 2020-05-26

**Authors:** Shintaro Shirahama, Atsuko Miki, Toshikatsu Kaburaki, Nobuyoshi Akimitsu

**Affiliations:** ^1^Department of Ophthalmology, Graduate School of Medicine, The University of Tokyo, Tokyo, Japan; ^2^Isotope Science Center, The University of Tokyo, Tokyo, Japan; ^3^Department of Ophthalmology, Jichi Medical University Saitama Medical Center, Saitama, Japan

**Keywords:** long non-coding RNA, immune response, infection, bacteria, virus

## Abstract

Recently developed technologies have revealed that the genomes of many organisms produce transcripts that do not encode proteins. These are called non-coding RNAs. Long non-coding RNAs (lncRNAs) are important regulators of the expression of their target genes at the levels of transcription, translation, and degradation. Multiple studies have demonstrated a role for lncRNAs in various biological responses, including pathogenic infection. Upon pathogenic infection, the expression levels of lncRNAs are dynamically altered, suggesting that lncRNAs are involved in the host immune response or propagation of pathogens. In this review, we focused on host lncRNAs that are involved in pathogenic infection. Some host lncRNAs act as host defense molecules to prevent pathogenic proliferation, while others are utilized by the pathogen to enhance the propagation of pathogens.

## Introduction

Recent transcriptome analyses using next-generation sequencing have revealed that the genomes of many species produce a large number of RNAs with low protein-coding potential, known as non-coding RNAs (ncRNAs). These ncRNAs were originally considered junk RNAs with no cellular functions; however, later studies have demonstrated that many of these ncRNAs are functional (Rinn and Chang, 2012).

Infectious diseases are caused by pathogens, including bacteria, viruses, fungi, or other parasites. The invasion of these pathogens often induces an inflammatory (Mogensen, 2009) or immune response of the host cell through the interferon pathway (Haller et al., 2006). In this review, we focus on bacteria and viruses. Although both (though not all bacteria) can proliferate inside the host cells, the mechanisms are different. As for bacteria, they proliferate in distinct compartments like organelles of the host cells. In contrast, viruses replicate themselves and are packaged within the host cells; that is, they can directly interact their host DNAs, RNAs, and proteins. Therefore, viruses can modulate or even hijack their host gene expression processes or metabolic pathways for their proliferation. For example, influenza A virus (IAV) robs host mRNAs of capped 5′ RNA nucleotides for efficient transcription of viral transcripts, a process called cap-snatching (Dias et al., 2009).

Although the protein network involved in protection against pathogen infection has been intensively investigated (de Chassey et al., 2008), RNA-mediated regulation remains largely unknown. Recent studies have examined the miRNA-regulated response against pathogen infection. MiRNAs of both host cells and viruses regulate each other’s gene expression by binding their target transcripts using seed sequences (reviewed in Skalsky and Cullen, 2010; Bruscella et al., 2017). However, little is known about lncRNA-mediated regulatory mechanisms during infection.

In this review, lncRNAs are defined as transcripts longer than 200 nucleotides with no protein-coding potential. Here we reviewed the lncRNAs involved in pathogen infection, especially bacteria and viruses, with a focus on host lncRNAs. These lncRNAs are either upregulated or downregulated during infection and can function in enhancing the host defense program or promoting pathogen invasion or replication within the host cells.

## Transcriptional Changes of LncRNAs Upon Pathogenic Infection

Transcriptome analyses revealed that lncRNAs as well as mRNAs show differential expression patterns during pathogen infection. For example, in response to meningitic *Escherichia coli* strain PCN033 infection of primary human brain microvascular endothelial cells, 382 lncRNAs were significantly upregulated and 513 were significantly downregulated (Yang et al., 2016). In WI-38 cells, antisense RNAs, a type of lncRNA expressed from the opposite strand of coding genes, are induced genome-widely after herpes simplex virus-1 (HSV-1) infection (Wyler et al., 2017). Another study revealed that 145 lncRNAs, including enhancer RNAs, were stabilized after *Salmonella enterica* serovar typhimurium virulent strain χ3306 infection, and knockdown of lncRNA NEAT1v2 or enhancer RNA eRNA07573 decreased cell survival rates after *Salmonella* infection, thus raising the possibility that these lncRNAs positively affect cell survival against *Salmonella* infection (Imamura et al., 2018). Together, these studies demonstrate that lncRNA expression levels are responsive to pathogenic infection.

## LncRNAs That Regulate Host Immune Response to Pathogenic Infection (Table 1)

There are several types of lncRNA-mediated host defense responses during infection. In this section, we focus on the lncRNAs involved in host defense by regulating immune-related genes.

**TABLE 1:** Long non-coding RNAs (LncRNAs) that regulate host immune response to pathogenic infection.

LncRNAs	Stimulation	Target genes (regulation)	Mechanism	References
IL1β-eRNA IL1β-RBT46	Lipopolysaccharide	*IL-1β* (↑), *CXCL8* (↑)	Unknown mechanism	IIott et al., 2014

Cox2	Lipopolysaccharide	*Ccl5* (↓), *Ip10* (↓), *Ptgs2* (↓), *Il6* (↑), *Il17* (↑)	Binds with heterogeneous nuclear ribonucleoprotein A/B and A2/B1	Carpenter et al., 2013; Elling et al., 2018

LncISG15 lncBST2/BISPR	IFNα type 2 Hepatitis C virus	*BST2* (↑)	Unknown mechanism	Barriocanal et al., 2014

NeST	Theiler’s virus	*IFN-γ* (↑)	Binds with WD repeat-containing protein 5 (WDR5) which is a component of the H3 lysine 4 methyltransferase complex	Gomez et al., 2013

CD244	Tuberculosis	*IFN-γ* (↓), *TNF-α* (↓)	Recruits polycomb protein enhancer of zeste homolog 2 (EZH2) and enhances H3K27 trimethylation at promoter regions	Wang et al., 2015

NRAV	Influenza A virus Sendai virus Muscovy Duck Reovirus Herpes simplex virus	*ISGs* (↓)	Decrease H3K4me3 at ISGs transcription start sites	Ouyang et al., 2014

LUARIS	poly (I:C)	*ISGs* (↓)	Directly interacts with heterogeneous nuclear ribonucleoprotein U (hnRNPU) and activating transcription factor 2 (ATF2)	Nishitsuji et al., 2016

NEAT1	(1) Influenza virus, Herpes simplex virus (2) Hantaan virus (3) DNA viruses	(1) *IL8* (↑) (2) *RIG-I* (↑), *DDX60* (↑), *IFN-β* (↑) (3) *IFN-α* (↑), *IFN-β* (↑)	(1), (2) Sequesters splicing factor proline and glutamine rich to paraspeckles (3) Interacts with HEXIM1 which was a transcription inhibitor	(1) Imamura et al., 2014 (2) Ma et al., 2017 (3) Morchikh et al., 2017

*BST2, bone marrow stromal cell antigen 2, CCL, C-C motif ligand, CXCL, C-X-C motif chemokine, DDX60, DExD/H-Box Helicase 60, IL, interleukin, IFN, interferon, ISGs, interferon-stimulated genes, Ptgs2, prostaglandin-endoperoxide synthase 2, RIG-I, retinoic acid-inducible gene-I.*

In human primary monocytes, stimulation by lipopolysaccharide (LPS), which is a main component of the outer membrane of Gram-negative bacteria, induces lncRNAs, enhancer RNAs, and bidirectional transcription. The *IL1β* locus is surrounded by enhancer RNA IL1β-eRNA and the bidirectionally transcribed transcript IL1β-RBT46. These lncRNAs localize in the nucleus and their expressions are dependent on NF-κB. Both IL1β-eRNA and IL1β-RBT46 regulate LPS-induced transcription of *IL1β* and *CXCL8* (IIott et al., 2014).

In CD11c positive bone-marrow-derived dendritic cells, the long intervening non-coding RNA (lincRNA)-Cox2 is an important regulator that is induced during LPS stimulation (Guttman et al., 2009). In mouse bone-marrow derived macrophages, the lincRNA-Cox2 binds with heterogeneous nuclear ribonucleoprotein A/B and A2/B1 to induce and repress genes involved in the inflammation response (Carpenter et al., 2013). Recently, functions of lincRNA-Cox2 were identified using lincRNA-Cox2 KO mice, and this lncRNA regulates its neighbor gene *Ptgs2 in cis* and inflammatory genes *in trans* (Elling et al., 2018). In HD11 cells, LPS stimulation initiates the transient synthesis of a non-coding RNA which is transcribed from the upstream region of the lysozyme gene, and this leads chromatin to an open conformation and thus activates lysozyme expression (Lefevre et al., 2008).

In HuH7 cells derived from human hepatocarcinoma, both lncISG15 and lncBST2/BISPR were identified upon IFNα2 treatment and were also induced by hepatitis C virus infection. These lncRNAs are induced by interferon-dependent pathway and expressed near interferon-stimulated gene loci. Knockdown of lncBST2/BISPR by siRNA leads to a reduction of BST2 expression, suggesting that this lncRNA has some roles in regulating the expression of its neighboring genes (Barriocanal et al., 2014).

**FIGURE 1:**
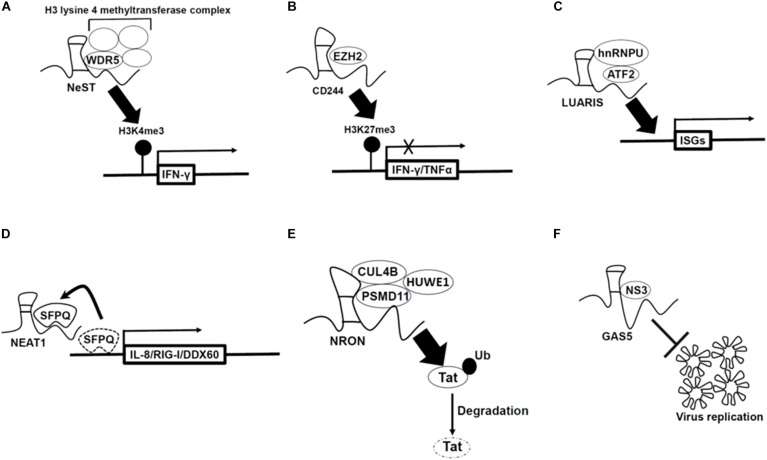
Mechanism by which long non-coding RNAs (lncRNAs) regulate antiviral genes expression **(A–D)** and viral replication **(E,F)**. **(A)** LncRNA-Nest promotes IFN-γ gene expression by binding to WD repeat-containing protein 5 (WDR5) which is a component of the H3 lysine 4 methyltransferase complex. **(B)** LncRNA-CD244 recruits enhancer of zeste homolog 2 (EZH2) polycomb protein which is H3K27 methyltransferase, and thus enhances H3K27 trimethylation at promoter regions of IFN-γ/TNF-α gene and inhibits gene expression. **(C)** LncRNA-LUARIS recruits transcription factors [heterogeneous nuclear ribonucleoprotein U (hnRNPU), activating transcription factor 2 (ATF2)], and thus binds to promoter region of ISGs and promotes gene expression. **(D)** NEAT1 sequesters SFPQ proteins which suppress immune-related genes under non-infected condition, and thus increases those gene expression. **(E)** LncRNA-NRON binds with ubiquitin ligase (CUL4B, PSMD1, and HUWE1) and then ubiquitylates Tat protein (viral transcription activator) to degrade, which inhibits viral replication. **(F)** LncRNA-GAS5 acts as decoy of hepatitis C virus non-structural protein 3 (NS3), which inhibits viral replication and assembly.

Some lncRNAs regulate gene expression of immune responsive genes by interacting with histone modification enzymes. For example, Nest is an lncRNA gene located adjacent to the interferon (IFN)-γ-encoding gene in both mice and human. In both genomes, NeST RNA is encoded on the DNA strand opposite to that coding for IFN-γ, and the two genes are transcribed by convergently (Vigneau et al., 2001). This lncRNA binds to WDR5, which is a component of the H3 lysine 4 methyltransferase complex, thus increasing expression of the *IFN-γ* locus (Figure 1A and Gomez et al., 2013). In CD8 positive T cells, tuberculosis infection-induced lncRNA-CD244 recruits enhancer of zeste homolog 2 (EZH2) polycomb protein and enhances H3K27 trimethylation at promoter regions of IFN-γ/TNF-α gene, thus downregulating these gene expression (Figure 1B and Wang et al., 2015). The lncRNA NRAV is suppressed upon IAV, Sendai virus, Muscovy Duck Reovirus, and a herpes simplex virus (HSV) infection to human alveolar epithelial cells. By overexpressing NRAV lncRNAs, the authors found that ISGs such as IFITM3 and MxA are downregulated with a decrease of H3K4me3 at their transcription start sites. In summary, NRAV acts as a negative regulator of ISGs (Ouyang et al., 2014).

Long non-coding RNA-LUARIS was identified by microarray analysis after treating human hepatocytes with poly(I:C), which mimics viral double-stranded RNAs. LUARIS is an antisense transcript of the protein-coding gene *HECW1* and is suppressed by type I interferon signaling. Knockdown of LUARIS by siRNA led to a reduction of ISGs expression. LUARIS directly interacts with heterogeneous nuclear ribonucleoprotein U (hnRNPU) and activating transcription factor 2 (ATF2) to regulate expressions of ISGs (Figure 1C and Nishitsuji et al., 2016).

The lncRNA NEAT1, an architectural non-coding RNA that forms paraspeckles, is also involved in antiviral response. NEAT1 expression is upregulated upon influenza virus and HSV infection in HeLa cells. Splicing factor proline- and glutamine-rich (SFPQ) binds to the promoter region of IL8 in non-infected cells. However, after poly(I:C) treatment which is mimicking of viral infection, NEAT1 is induced and sequesters SFPQ to paraspeckles, thus enabling the expression of antiviral genes including those encoding cytokines such as IL-8 (Figure 1D and Imamura et al., 2014). A similar mechanism was also reported upon infection of Hantaan virus. In human umbilical vein endothelial cells, activation of the RIG-I-IRF7 pathway by Hantaan virus infection induced transcription of the *NEAT1* gene. Then, NEAT1 sequesters SFPQ to paraspeckles to induce *RIG-I* and *DDX60* transcription, thus leading to interferon-β production (Ma et al., 2017). NEAT1 also interacts with HEXIM1 which was previously discovered as a transcription inhibitor. In HeLa cells, NEAT1-HEXIM1 complex regulates the immune response through the cGAS-STING pathway upon infection by DNA viruses such as Kaposi’s sarcoma-associated herpesvirus (Morchikh et al., 2017).

## LncRNAs That Inhibit Pathogen Proliferation (Table 2)

Another mechanism for lncRNA function in host defense is the prevention of pathogen replication. For example, in Jurkat cells, the lncRNA NRON represses human immunodeficiency virus type 1 (HIV-1) replication by inhibiting the transcription factor nuclear factor of activated T cells (NFAT) which enhances viral replication of HIV-1 (Imam et al., 2015). Recent studies showed that lncRNA NRON degrades Tat protein (viral transcription activator) through ubiquitination by interacting with the ubiquitin ligase CUL4B, PSMD11, and HUWE1 (UREB1) in TZM-bl cells (Figure 1E and Li et al., 2016).

**TABLE 2:** Long non-coding RNAs (LncRNAs) involved in pathogen proliferation.

LncRNAs that inhibit pathogen proliferation	Target pathogen	Mechanism	References
NRON	HIV-1	(1) Inhibits the transcription factor nuclear factor of activated T cells (NFAT) which enhances viral replication of HIV-1 (2) Degrades the viral transcription activator Tat protein through ubiquitination by interacting with the ubiquitin ligase	(1) Imam et al., 2015 (2) Li et al., 2016

GAS5	Hepatitis C virus	Decoy of the hepatitis C virus non-structural protein 3 (NS3)	Qian et al., 2016

NEAT1	HSV-1 HIV-1	(HSV-1) Directly interact with HSV-1 genomes and modulate virus gene expression (HIV-1) Downregulates non-spliced form of HIV-1 mRNAs that contain instability elements	Zhang et al., 2013 Zhang et al., 2013

**LncRNAs that promote pathogen proliferation**			

VIN	Influenza A virus	Unknown mechanism	Winterling et al., 2014

PAAN	Influenza A virus	Helps the formation of the viral RNA polymerase complex by interacting with its component polymerase acidic protein	Wang et al., 2018

ACOD1	Vesicular stomatitis virus	Directly interacts with the metabolic enzyme glutamic-oxaloacetic transaminase to enhance its catalytic activity	Wang P. et al., 2017

EGOT	Hepatitis C virus	Unknown mechanism	Carnero et al., 2016

*HIV-1, human immunodeficiency virus type 1, HSV-1, herpes simplex virus type 1.*

Previously annotated lncRNAs have also been identified as repressors of viral replication. LncRNA GAS5, which was first identified as a growth-arrest specific transcript (Schneider et al., 1988) and related to cancer (Pickard et al., 2013), prevents hepatitis C virus replication in Huh7 cells. In addition, this lncRNA acts as a decoy of the hepatitis C virus NS3 protein which is important for viral replication and assembly (Figure 1F and Qian et al., 2016).

The lncRNA NEAT1 represses HIV-1 replication. HIV-1 mRNAs are spliced and exported to the cytoplasm through a Rev-dependent pathway (Malim et al., 1989). When NEAT1 is downregulated using siRNA, in HeLa cells, the non-spliced form of HIV-1 mRNAs that contain instability elements are found in the cytoplasm and leads to increased HIV-1 replication (Zhang et al., 2013). Similarly, in HeLa cells, NEAT1 and protein components of paraspeckles directly interact with HSV-1 genomes, and modulate both viral gene expression and replication of HSV-1 through the STAT3 transcription factor (Wang Z. et al., 2017).

## LncRNAs That Promote Pathogen Proliferation (Table 2)

Some host lncRNAs are used by pathogens to promote pathogen proliferation. For example, lncRNA VIN was identified by microarray analysis in human lung epithelial cells infected with IAV. Downregulation of the lncRNA VIN by siRNA reduced IAV replication, thus indicating that this lncRNA is involved in efficient proliferation of IAV in host cells (Winterling et al., 2014). The lncRNA PAAN, which is upregulated upon IAV infection in HEK293T cells, helps the formation of the viral RNA polymerase complex by interacting with its component PA protein (Wang et al., 2018). The lncRNA ACOD1 is upregulated upon infection by viruses such as vesicular stomatitis virus, Sendai virus, HSV-1, and Vaccinia virus in mouse pertitoneal macrophages. Transcription of the lncRNA ACOD1 is independent of type-1 interferon but is induced by the NF-κB pathway which is activated by viral infection. The lncRNA ACOD1 directly interacts with the metabolic enzyme glutamic-oxaloacetic transaminase to enhance its catalytic activity, thus leading to viral proliferation (Wang P. et al., 2017). The lncRNA EGOT is increased by infection of hepatitis C virus, influenza virus or Semliki Forest virus through RIG-I and PKR activation in HuH7 cells. Downregulation of EGOT resulted in a decrease of hepatitis C virus replication. By performing guilt-by-association analysis, it was predicted that EGOT is negatively correlated with innate immune responsive genes such as *TLR3*, *NF-κB*, and *IRF3*. Therefore, the authors suggested that EGOT regulates the antiviral pathway negatively (Carnero et al., 2016).

## Concluding Remarks and Perspectives

Long non-coding RNAs have attracted lots of attention because they are involved in various cellular functions despite their “non-coding” nature. Among them, some lncRNAs are identified as important regulators of host response toward pathogen infection as described in this review. However, considering that the functions of lncRNAs are regulated spatio-temporally, these analyses have dismissed the time-dependent effect or localization-dependent function of lncRNAs. Therefore, it might be possible that there are unknown lncRNAs whose expression levels do not change during infection but function by changing their localization patterns. In addition, NGS analyses using bulk (mixed population) samples disregard the heterogeneity of host cells or the difference of infection stages of the host cells, which results in difficulty in identifying functional lncRNAs whose expression levels differ among each host cell. The recently developed single cell RNA-seq (scRNA-seq) approach for infected cells is more informative (reviewed in Cristinelli and Ciuffi, 2018); however, scRNA-seq also is limited in its ability to obtain sufficient read depths of rarely expressed RNAs, such as lncRNAs or pathogen-derived RNAs.

The development of new technologies to overcome the limitations of read-depths or methods for detecting changes of RNA localization will identify new aspects of lncRNA-mediated responses toward pathogen infection.

## Author Contributions

All authors listed have made a substantial, direct and intellectual contribution to the work, and approved it for publication.

## Conflict of Interest

The authors declare that the research was conducted in the absence of any commercial or financial relationships that could be construed as a potential conflict of interest.
